# Differences in Birth Weight Associated with the 2008 Beijing Olympics Air Pollution Reduction: Results from a Natural Experiment

**DOI:** 10.1289/ehp.1408795

**Published:** 2015-04-28

**Authors:** David Q. Rich, Kaibo Liu, Jinliang Zhang, Sally W. Thurston, Timothy P. Stevens, Ying Pan, Cathleen Kane, Barry Weinberger, Pamela Ohman-Strickland, Tracey J. Woodruff, Xiaoli Duan, Vanessa Assibey-Mensah, Junfeng Zhang

**Affiliations:** 1 Department of Public Health Sciences, School of Medicine and Dentistry, University of Rochester, Rochester, New York, USA; 2 Department of Maternal and Child Health, Beijing Obstetrics and Gynecology Hospital, Capital Medical University, Beijing, China; 3 Department of Environmental Pollution and Health, Chinese Research Academy of Environmental Health Sciences, Beijing, China; 4 Department of Biostatistics and Computational Biology, and; 5 Department of Pediatrics, School of Medicine and Dentistry, University of Rochester, Rochester, New York, USA; 6 Department of Pediatrics, Robert Wood Johnson Medical School, Rutgers University, New Brunswick, New Jersey, USA; 7 Department of Biostatistics, School of Public Health, Rutgers University, Piscataway, New Jersey, USA; 8 Program on Reproductive Health and the Environment, Department of Obstetrics, Gynecology and Reproductive Sciences, University of California San Francisco, San Francisco, California, USA; 9 Nicholas School of the Environment, and; 10 Duke Global Health Institute, Duke University, Durham, North Carolina, USA

## Abstract

**Background:**

Previous studies have reported decreased birth weight associated with increased air pollutant concentrations during pregnancy. However, it is not clear when during pregnancy increases in air pollution are associated with the largest differences in birth weight.

**Objectives:**

Using the natural experiment of air pollution declines during the 2008 Beijing Olympics, we evaluated whether having specific months of pregnancy (i.e., 1st…8th) during the 2008 Olympics period was associated with larger birth weights, compared with pregnancies during the same dates in 2007 or 2009.

**Methods:**

Using *n* = 83,672 term births to mothers residing in four urban districts of Beijing, we estimated the difference in birth weight associated with having individual months of pregnancy during the 2008 Olympics (8 August–24 September 2008) compared with the same dates in 2007 and 2009. We also estimated the difference in birth weight associated with interquartile range (IQR) increases in mean ambient particulate matter ≤ 2.5 μm in aerodynamic diameter (PM_2.5_), sulfur dioxide (SO_2_), nitrogen dioxide (NO_2_), and carbon monoxide (CO) concentrations during each pregnancy month.

**Results:**

Babies whose 8th month of gestation occurred during the 2008 Olympics were, on average, 23 g larger (95% CI: 5 g, 40 g) than babies whose 8th month occurred during the same calendar dates in 2007 or 2009. IQR increases in PM_2.5_ (19.8 μg/m^3^), CO (0.3 ppm), SO_2_ (1.8 ppb), and NO_2_ (13.6 ppb) concentrations during the 8th month of pregnancy were associated with 18 g (95% CI: –32 g, –3 g), 17 g (95% CI: –28 g, –6 g), 23 g (95% CI: –36 g, –10 g), and 34 g (95% CI: –70 g, 3 g) decreases in birth weight, respectively. We did not see significant associations for months 1–7.

**Conclusions:**

Short-term decreases in air pollution late in pregnancy in Beijing during the 2008 Summer Olympics, a normally heavily polluted city, were associated with higher birth weight.

**Citation:**

Rich DQ, Liu K, Zhang J, Thurston SW, Stevens TP, Pan Y, Kane C, Weinberger B, Ohman-Strickland P, Woodruff TJ, Duan X, Assibey-Mensah V, Zhang J. 2015. Differences in birth weight associated with the 2008 Beijing Olympics air pollution reduction: results from a natural experiment. Environ Health Perspect 123:880–887; http://dx.doi.org/10.1289/ehp.1408795

## Introduction

Previous studies have examined the association between exposure to air pollution at various times during pregnancy (e.g., first, second, or third trimester or whole pregnancy) and birth weight, but have reported inconsistent findings partly because of differences in study design (time series, cohort study), study data sources (birth certificates vs. hospital discharge data), exposure error related to pollution data (e.g., from central-site monitors, land use regression estimates), and potential residual confounding by subject characteristics (Woodruff et al. 2009). In a meta-analysis of 14 studies, increased concentrations of particulate matter with aerodynamic diameter ≤ 10 μm (PM_10_) and ≤ 2.5 μm (PM_2.5_) across the entire pregnancy were associated with increases in the risk of low birth weight (< 2,500 g) and decreases in birth weight among term births (≥ 37 weeks gestation) (Dadvand et al. 2013). However, because these studies did not identify specific gestational age windows (e.g., early or late pregnancy) during which exposure to air pollution was consistently associated with low birth weight, understanding potential mechanisms of any pollutant effect on fetal growth has been difficult (Woodruff et al. 2009).

Within natural experiments, where an industrial employee strike, legislative mandate, or large-scale sporting event resulted in decreased pollutant concentrations or changes in the air pollution composition over a city or region, investigators have reported reductions in respiratory outcomes (Friedman et al. 2001; Heinrich et al. 2000; Lee et al. 2007; Pope 1989, 1991), total and/or cause-specific mortality rates (Clancy et al. 2002; Hedley et al. 2002; Pope et al. 2007), and increases in life expectancy (Tonne et al. 2008). Using data from the closure of a large steel mill in the Utah Valley for 13 months (Pope 1989, 1991), when PM_10_ concentrations were reduced by about 43% (from 90 μg/m^3^ to 51 μg/m^3^) (Pope 1989), we previously reported that mothers who were already pregnant at the time of mill closure were less likely to deliver prematurely than mothers pregnant before or after the closure. Further, the strongest associations were observed for pregnancies in which the second trimester occurred during the mill closure [relative risk (RR) = 0.86; 95% confidence interval (CI): 0.75, 0.98]. However, we did not find a significant reduction in birth weight for babies born during the steel mill closure (Parker et al. 2008). Although this study took advantage of a natural experiment to examine changes in preterm birth and fetal growth, the duration of the reduction in air pollution was relatively long (~ 13 months), thus making investigation into the gestational age–specific windows of pregnancy (e.g., first month or last month), when reduced air pollution might result in improved pregnancy outcomes, difficult.

Beijing is one of the most polluted cities in the world, with annual mean concentrations of PM_2.5_ exceeding 100 μg/m^3^ (Zhang et al. 2010) and daily mean concentrations of PM_2.5_ at times exceeding 200 μg/m^3^ (Xu and Zhang 2004), with several studies documenting even higher levels on “haze days” compared with “nonhaze” days (Sun et al. 2013; Tao et al. 2014; Zhao et al. 2013). Sources contributing to Beijing’s air pollution are complex, but include local emissions from motor vehicles, residential/commercial/industrial combustion devices, and fugitive emissions, as well as long-range transport of regional pollutants and secondary formation (Wang et al. 2010; Zhou et al. 2010). Thus, as a condition for hosting the 2008 Olympic Games, the Chinese government agreed to temporarily and substantially improve air quality in Beijing for the Olympics and subsequent Paralympics (8 August to 24 September 2008). These actions included implementing heightened vehicular emissions standards, restricting use of vehicles by license plate number, relocating and closing industrial facilities in Beijing and the surrounding province, and stopping construction activities (Zhang et al. 2013). We previously reported that concentrations of several pollutants [PM_2.5_, nitrogen dioxide (NO_2_), carbon monoxide (CO), sulfur dioxide (SO_2_), sulfate, elemental carbon, organic carbon] monitored between June and October of 2008 decreased during this Olympics/Paralympics period by 18–59% from pre-Olympics levels (Rich et al. 2012; Zhang et al. 2013). In fact, daily concentrations of PM_10_, NO_2_, and SO_2_ all gradually decreased in the weeks preceding the Olympics and also increased gradually in the weeks following the Olympics. Although these were general trends, large day-to-day variations in pollutant levels were observed during each of the pre-, during-, and post-Olympics periods, largely due to variations in meteorological conditions (Zhang et al. 2013).

Using birth records from mothers residing in Beijing during their pregnancies, we hypothesized that term pregnancies (i.e., 37–41 weeks gestational age) with at least 1 month of pregnancy during the Olympics/Paralympics period would have higher birth weights than pregnancies with those same gestational months during 8 August–24 September 2007 or 8 August–24 September 2009 (hypothesis 1). Further, using our air pollution data from 2 June to 30 October 2008, we hypothesized that increased air pollution concentrations would be associated with decreased birth weights among term births in pregnancies with at least 1 month of pregnancy (months 1–8) occurring between 2 June and 30 October 2008 (hypothesis 2). Our first goal was to evaluate whether short periods of pregnancy during the time of lowered overall air pollution levels would be associated with increased birth weight. Our second goal evaluated whether specific air pollutants were associated with these birth weight changes.

## Methods

*Study population and birth data*. Records of Beijing births are created and maintained by the Beijing Obstetrics and Gynecology Hospital. Using this birth registry, we included all singleton live births of infants who had ≥ 28 completed weeks of gestation occurring between 1 January 2007 and 31 December 2010 to mothers who resided in one of four adjacent Beijing districts (Xicheng, Haidan, Fengtai, and Chaoyang) at the time of birth (*n* = 140,298). Gestational age was calculated as the number of complete weeks since the end of the last menstrual period. For each birth/pregnancy, we also obtained the following variables: birth weight (grams), maternal age at birth (years), gestational week of first medical checkup during pregnancy, maternal occupation, maternal education level, pregnancy complications (gestational hypertension, preeclampsia, eclampsia, fetal macrosomia, fetal distress, placental abruption, threatened preterm labor, polydramnios, oligohydramnios, and premature rupture of the membranes), and whether the pregnancy resulted in a live birth or stillbirth. Gestational hypertension was defined as systolic blood pressure ≥ 140 mmHg, diastolic blood pressure ≥ 90 mmHg, or both, on two or more occasions at least 4 hr apart in normotensive women after 20 weeks gestation. Preeclampsia was defined as new-onset hypertension, in addition to new-onset proteinuria, or in the absence of proteinuria, new-onset end-organ dysfunction after 20 weeks gestation (e.g., thromobocytopenia, renal insufficiency, impaired liver function, pulmonary edema, or cerebral or visual symptoms). Eclampsia was defined as preeclampsia with grand mal seizures. Fetal macrosomia was defined as a birth weight > 4,000 g regardless of gestational age. Fetal distress was defined as the presence of signs or symptoms that the fetus was not well, including decreased movement felt by the mother, meconium in the amniotic fluid, a nonreassuring fetal heart rate pattern, or fetal acidosis. Placental abruption was defined as premature separation of a normally implanted placenta from the uterus after 20 weeks gestation. Threatened preterm labor was defined as documented uterine contractions without evidence of cervical change. Preterm labor was defined as documented uterine contractions associated with evidence of cervical change, with the delivery ended before 37 weeks gestation. Polyhydramnios was defined as excess amniotic fluid volume (AFV) or index (AFI) (i.e., AFV ≥ 8 cm or AFI ≥ 25 cm), whereas oligohydramnios was defined as amniotic fluid volume less than expected for gestational age (AFV ≤ 2 cm or AFI ≤ 5 cm). Premature rupture of membranes was defined as rupture of the amniotic sac before labor began.

We then excluded births with weights more or less than 5 standard deviations above or below the mean (*n* = 73), and those with a gestational age at birth of > 41 weeks (*n* = 1,575), so as to avoid errors in gestational age and birth weight. We did not have data on the infants’ sex or mode of delivery (vaginal or cesarean). After deleting 5,809 births with dates of last menstrual periods before 19 June 2006 and after 31 March 2010 to avoid fixed cohort bias (because we were studying both preterm birth and birth-weight effects of air pollution) (Barnett 2011), we then excluded all preterm deliveries (*n* = 4,937 with < 37 weeks gestational age), leaving 127,904 term births (37–41 weeks gestational age) available for analysis. This study was approved by the Research Subjects Review Board at the University of Rochester Medical Center and the Ethics Review Committee of Peking University Health Sciences Center in Beijing, China.

*Air pollution and weather*. First, we obtained daily PM_10_, NO_2_, and SO_2_ concentrations averaged across the air pollution monitoring stations located in the same four Beijing districts (1,258 km^2^) in which our study subjects lived from 8 August to 24 September in 2007, 2008, and 2009. We then used these daily Beijing-average concentrations to describe the differences in mean pollutant concentrations observed in 2007, 2009, and 2008 during the Olympics for descriptive purposes only (hypothesis 1).

Second, we used our previously collected air pollution and weather measurements, which included PM_2.5_ and CO (Rich et al. 2012; Zhang et al. 2013), in our hypothesis 2 statistical analyses. Briefly, we measured hourly concentrations of fine particles (PM_2·5_) using a tapered element oscillating microbalance (TEOM) from 2 June to 30 October 2008. We also measured gaseous pollutants (SO_2_, NO_2_, CO) during these same times using monitors that were calibrated and maintained following manufacturer’s protocols (Ecotech Ltd., Knoxfield, VIC, Australia). We measured ambient temperature and relative humidity (RH) at the same site. All the samplers and monitors were collocated on the rooftop of a seven-story building in the center of the Peking University First Hospital campus, located in central Beijing. The First Hospital pollutant concentrations were well correlated with the Beijing average pollutant concentrations (NO_2_: *r* = 0.84; SO_2_: *r* = 0.65; PM_10_ vs. PM_2.5_: *r* = 0.85). These pollutant and weather data were used in our statistical analyses for hypothesis 2 described below.

*Statistical analyses*. Hypothesis 1. First, using the date of birth and gestational age included in the data set, we calculated the beginning and end of each month of pregnancy. We then included only those pregnancies ending in a term birth with at least 24 days of pregnancy in a given month (i.e., either 1st, 2nd, 3rd … 8th month) occurring in the 47 days between 8 August and 24 September in 2007, 2008, or 2009. This definition ensures that any subject has at most 1 month of pregnancy in the Olympics period in our analysis. For simplicity of presentation, we defined the 1st month of pregnancy to begin with the 1st day after the last menstrual period. No attempt was made to correct for the date of conception. Using these 71,803 births, we calculated descriptive statistics for subject characteristics by year. In preparation for model fitting, using all 71,803 term births, we fit a semiparametric additive model (Hastie and Tibshirani 1990) in which birth weight was modeled as a smooth function of maternal age, adjusting for indicator variables for the Olympics (i.e., 2008 vs. 2007 and 2009), residential district, gestational week, and maternal education level (bachelor’s degree, some college or technical school, high school or less). We used both 2007 and 2009 as controls to control for confounding by time trends in birth weight and air pollution across these years. Using generalized cross-validation [using the mgcv package in R (R Core Team 2014)] we determined that 4 degrees of freedom for the smooth on maternal age was reasonable.

Next, we fit eight separate semiparametric additive models, one for each month of pregnancy (months 1–8 only), in which birth weight was modeled as a smooth function of maternal age with 4 degrees of freedom. We then re-ran these analyses including the covariates described above. The smooth function was estimated by a smoothing spline, using the gam package in R. We used results from these models to estimate the difference in birth weight among term births (with 95% CI) associated with having a specified month of pregnancy during the 2008 Olympics period, compared with having that same month of pregnancy during the same dates in 2007 or 2009.

Hypothesis 2. Starting from the 127,904 births, we included only term births with at least 1 month of pregnancy (months 1–8) occurring between 2 June and 30 October 2008 (time period for which we measured the air pollutants described above), leaving 32,506 births (83,672 observations; subjects could have multiple months of pregnancy in this time period) available for analysis. We calculated descriptive statistics for air pollutant concentrations, weather conditions, and subject characteristics for these births. We then calculated mean pollutant concentrations, temperature, and relative humidity for each gestational month for all months where at least 75% of the measured values were not missing. Again using semiparametric additive models, we regressed birth weight on the mean PM_2.5_ concentration in the 1st month of pregnancy for each study subject. We also included indicator variables for gestational age (complete weeks) at delivery, residential district, maternal education (bachelor’s degree, some college or technical school, high school or less), linear terms for the mean temperature and relative humidity levels during the same 1st month of pregnancy, and a smooth term for maternal age (smoothing spline with 4 degrees of freedom). We repeated this same model to estimate the difference in birth weight associated with the mean 1st month SO_2_, NO_2_, and CO concentrations. We then repeated these analyses for the 2nd, 3rd … 8th month mean pollutant concentrations in the same manner.

Sensitivity analyses. To examine whether our findings were limited to pregnancies with one or more pregnancy complications (i.e., less healthy individuals), we restricted our analyses to those without a pregnancy-related hypertensive disorder (i.e., without gestational hypertension, preeclampsia, or eclampsia) and then again to those without a fetal placental condition (i.e., without fetal macrosomia, fetal distress, placental abruption, threatened preterm labor, polydramnios, oligohydramnios, or premature rupture of the membranes). We did not have data on preexisting hypertension. We then re-ran the same set of models described above. Next, for each year separately, we generated Q-Q plots of residuals from our hypothesis 1: 8th month model described above. In these plots, residuals were sorted, and the sorted values were plotted against the expected sorted birth weight values from a normal distribution with the same mean and variance. If the model residuals had an exact normal distribution, the points would fall on the diagonal 1:1 line. We were interested in deviations from normality in the extreme values (e.g., the upper and lower tails). If deviations from normality at the upper or lower tails differed for 2008 compared with 2007 or 2009, this would suggest differences in birth weight associated with the 2008 Olympics period were restricted to either small or large for gestational age babies. Last, we examined whether our findings were limited to pregnant women residing in only one Beijing district or only to women who were college graduates (i.e., presumably of higher socioeconomic status). We estimated the difference in birth weight associated with having a specific month of pregnancy during the 2008 Olympics period compared to the same calendar dates in 2007 or 2009, for pregnant women living in each district separately, by adding interaction terms (Fengtai × 2008; Haidan × 2008; Chaoyang × 2008) to these models. Similarly, we estimated these same birth weight differences for women with a bachelor’s degree or more, some technical school, or high school or less, separately adding interaction terms to the models (TechSchool × 2008; HighSchool × 2008). We used SAS version 9.32 (SAS Institute Inc., Cary, NC, USA) to construct all datasets and conduct descriptive analyses, and R (version 2.15.1) to perform all semiparametric additive model analyses.

## Results

**Figure 1:**
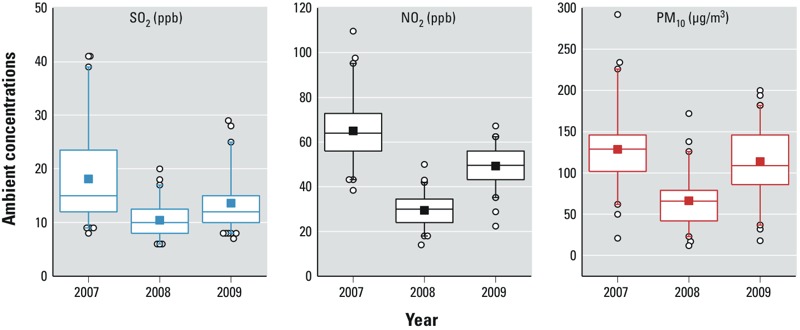
Distributions of daily mean SO_2_, NO_2_, and PM_10_ concentrations across Beijing from 8 August to 24 September 2007, 2008, and 2009. Boxes extend from the 25th to the 75th percentile, horizontal bars represent the median, whiskers indicate the 5th and 95th percentiles, squares indicate the mean, and circles represent outliers.

*Hypothesis 1*. Characteristics of the study subjects included in hypothesis 1 analyses (*n* = 70,787) are shown in Table 1. Across the 3 years (2007–2009), the distributions of maternal age, gestational age at delivery, residential district, frequency of pregnancy complications, and birth weight are similar. However, maternal education level increases slightly across the 3 years (percentage of mothers with bachelor’s degree increases from 50.8% in 2007 to 60.6% in 2009). Daily PM_10_, NO_2_, and SO_2_ concentrations were substantially lower in 2008 during the Olympics than during the same dates in 2007 or 2009 (Figure 1).

**Table 1:** Study subject characteristics by year [n (%) or mean ± SD unless otherwise indicated].

Characteristic	Control period 2007 (*n*=21,227)	Olympics period 2008 (*n*=23,361)	Control period 2009(*n*=26,201)
Maternal age (years)
<24	771 (3.6)	918 (3.9)	874 (3.3)
24–26	4,140 (19.5)	3,857 (16.5)	3,560 (13.6)
27–29	7,436 (35.0)	8,211 (35.1)	9,684 (37.0)
30–32	5,186 (24.4)	6,207 (26.6)	7,456 (28.5)
33–35	2,535 (11.9)	2,752 (11.8)	2,906 (11.1)
>35	1,159 (5.5)	1,416 (6.1)	1,721 (6.6)
Mean ± SD	29.2 ± 3.6	29.3 ± 3.6	29.5 ± 3.6
Maternal education
Bachelor	10,793 (50.8)	12,768 (54.7)	15,886 (60.6)
Some college or technical school	3,250 (15.3)	3,501 (15.0)	3,305 (12.6)
High school or less	7,184 (33.8)	7,092 (30.4)	7,010 (26.8)
Residential district
Chaoyang	7,971 (38)	9,399 (40)	11,111 (42)
Fentai	4,732 (22)	4,959 (21)	5,651 (22)
Haidian	6,852 (32)	7,537 (32)	7,845 (30)
Xicheng	1,672 (8)	1,466 (6)	1,594 (6)
Pregnancy complications
Pregnancy-related hypertensive disorders	156 (0.7)	236 (1.0)	291 (1.1)
Gestational hypertension	93 (0.4)	143 (0.6)	188 (0.7)
Preeclampsia	59 (0.3)	88 (0.4)	101 (0.5)
Eclampsia	4 (0.0)	5 (0.0)	2 (0.0)
Fetal–placental conditions	2,458 (11.6)	2,736 (11.7)	3,129 (11.9)
Fetal macrosomia	1,413 (6.7)	1,533 (6.6)	1,772 (6.8)
Fetal distress	511 (2.4)	613 (2.6)	648 (2.5)
Placental abruption	13 (0.1)	24 (0.1)	28 (0.1)
Threatened preterm labor	30 (0.1)	15 (0.1)	16 (0.1)
Polyhydramnios	22 (0.1)	20 (0.1)	42 (0.2)
Oligohydramnios	215 (1.0)	221 (0.9)	294 (1.1)
Premature rupture of membranes	400 (1.9)	457 (2.0)	527 (2.0)
Gestational age (weeks)
37	1,313 (6)	1,598 (7)	1,660 (6)
38	4,247 (20)	4,921 (21)	5,327 (20)
39	6,838 (32)	7,553 (32)	8,478 (32)
40	6,141 (29)	6,560 (28)	7,543 (29)
41	2,688 (13)	2,729 (12)	3,193 (12)
Mean ± SD	39.3 ± 1.1	39.2 ± 1.1	39.2 ± 1.1
Birth weight (g)
Mean ± SD	3,417 ± 414	3,414 ± 419	3,415 ± 410
Minimum	1,290	1,500	930
5th percentile	2,770	2,750	2,760
10th percentile	2,900	2,900	2,900
25th percentile	3,145	3,130	3,150
50th percentile	3,400	3,400	3,400
75th percentile	3,690	3,685	3,680
90th percentile	3,950	3,950	3,950
95th percentile	4,100	4,120	4,100
Maximum	5,250	5,800	5,860

In unadjusted analyses, having the 2nd month or the 8th month during the 2008 Beijing Olympics, compared with the same dates in 2007 or 2009, was associated with a significant decrease (21 g; 95% CI: –40 g, –2 g) and increase (22 g; 95% CI: 5 g, 40 g) in birth weight, respectively (Table 2). After adjusting for several covariates, pregnancies for which the 2nd month of gestation occurred during the 2008 Olympics period had a small, nonsignificantly decreased birth weight (–16 g; 95% CI: –345 g, 2 g) compared with pregnancies for which the 2nd month of gestation occurred during the same dates in 2007 or 2009. However, pregnancies having a month of gestation other than the 2nd month during the 2008 Olympics, instead of the same dates in 2007 or 2009, were generally associated with small increases in birth weight, with the largest being a significant 23-g increase (95% CI: 5 g, 40 g) in birth weight among those pregnancies with the 8th month during the 2008 Olympic games (Table 2).

**Table 2:** Difference in birth weight associated with a month of pregnancy during 2008 (8 August to 24 September 2008), compared with the same dates in 2007 or 2009.

Month of pregnancy^*a*^	*n*	Unadjusted analyses		Adjusted analyses	
		Difference in birth weight (g) (95% CI)	*p*-Value	Difference in birth weight (g) (95% CI)^*b*^	*p*-Value
1	8,969	2 (–16, 20)	0.81	4 (–13, 21)	0.62
2	8,305	–21 (–40, –2)	0.03	–16 (–35, 2)	0.08
3	8,191	–11 (–31, 9)	0.29	4 (–15, 24)	0.68
4	8,864	–14 (–32, 4)	0.13	–4 (–22, 14)	0.64
5	9,065	2 (–16, 20)	0.86	3 (–15, 21)	0.74
6	9,294	0 (–17, 18)	0.96	5 (–12, 22)	0.57
7	9,808	0 (–17, 17)	0.99	5 (–12, 22)	0.54
8	9,307	22 (5, 40)	0.01	23 (5, 40)	0.01
^***a***^We defined the 1st month of pregnancy to begin with the 1st day after the last menstrual period. ^***b***^All models included indicator variables for gestational age (complete weeks) at delivery, residential district, maternal education (bachelor’s degree, some college or technical school, high school or less), and a smooth term for maternal age (smoothing spline with 4 degrees of freedom).					

**Figure 2:**
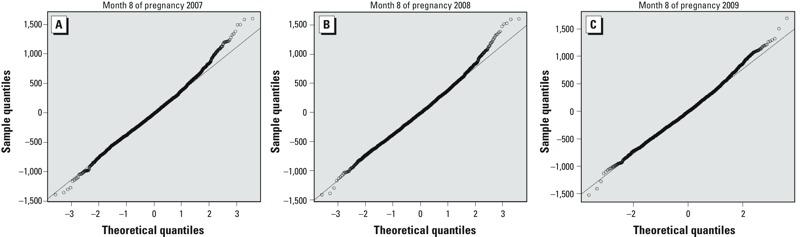
Plots of sorted birth weight residuals for births with the 8th month of pregnancy from 8 August to 24 September versus sorted expected values from a normal distribution with the same mean and variance (Q-Q plots) in (*A*) 2007, (*B*) 2008, or (*C*) 2009.

We next evaluated whether our 8th month finding was limited to pregnancies with one or more complications (i.e., either a pregnancy-related hypertensive disorder or a fetal placental condition), or to small or large birth weight babies. When excluding the 2.0% (*n* = 189) of pregnancies with a pregnancy-related hypertensive disorder and then the 12.9% (*n* = 1,204) of pregnancies with a fetal placental condition, having the 8th month of pregnancy during the 2008 Olympics period instead of the same dates in 2007 or 2009 was still associated with significant 24-g (95% CI: 7 g, 42 g) and 22-g (95% CI: 4 g, 37 g) increases in birth weight, respectively. Further, Q-Q plots revealed similar patterns of residuals in the upper and lower ends of the birth weight distribution across all 3 years (Figure 2). Thus, our reported increase in birth weight for pregnancies with their 8th month of pregnancy during the Olympics period (23 g), compared with the same dates in 2007 or 2009 described above, does not appear to be driven by differences in the low or high end of the birth weight distribution. We observed increases in birth weight associated with the 8th month of pregnancy occurring during the 2008 Olympics period, compared with the same dates in 2007 and 2009, among pregnant women living in Xicheng (20 g; 95% CI: –46 g, 86 g), Haidan (41 g; 95% CI: 10 g, 71 g), and Chaoyang (32 g; 95% CI: 4 g, 59 g) districts, but not in Fengtai (–18 g; 95% CI: –57 g, 19 g). Last, we observed increases in birth weight associated with the 8th month of pregnancy occurring during the 2008 Olympics period, compared with the same dates in 2007 and 2009, for all maternal education levels (bachelor’s degree or more: 29 g, 95% CI: 5 g, 52 g; technical school: 22 g, 95% CI: –9 g, 52 g; high school or less: 5 g; 95% CI: –40 g, 50 g), although the largest difference was observed among those with a bachelor’s degree or more.

*Hypothesis 2.* Moving average monthly pollutant concentrations and weather characteristics for the period of analysis (2 June to 30 October 2008) are shown in Table 3. Study subjects’ gestational month mean PM_2.5_ concentrations were highly correlated with both their SO_2_ (*r* = 0.86) and CO mean concentrations (*r* = 0.71), but slightly negatively correlated with NO_2_ concentrations (*r* = –0.25) (Table 4). Monthly mean NO_2_ concentrations were highly inversely correlated with both temperature (*r* = –0.96) and relative humidity means (*r* = –0.70). Monthly mean PM_2.5_ concentrations were moderately correlated with temperature (*r* = 0.41), but monthly mean SO_2_ and CO concentrations were not (*r* ≤ 0.10). Monthly mean PM_2.5_, SO_2_, and CO concentrations were only weakly correlated with monthly mean relative humidity levels (*r* ≤ 0.28).

**Table 3:** Descriptive statistics of mean ambient air pollutant concentrations and weather characteristics in Beijing, China, at First Hospital (2 June–30 October 2008; *n* = 11,770).

Pollutant/weather characteristic	*n*	Mean ± SD	Minimum	Percentile					Maximum
				5th	25th	50th	75th	95th	
Monthly averages^*a*^
Temperature (°C)	11,770	24.9 ± 3.8	16.8	17.4	22.1	25.9	27.8	29.1	29.6
Relative humidity (%)	11,770	62.4 ± 5.0	47.7	52.1	61.1	63.3	64.9	68.3	69.8
PM_2.5_ (μg/m^3^)	10,771	61.3 ± 11.1	43.7	46.4	51.5	59.8	71.3	77.8	85.1
SO_2_ (ppb)	8,961	5.9 ± 1.2	3.7	4.0	5.1	5.7	6. 9	7.5	8.2
NO_2_ (ppb)	11,770	24.3 ± 8.5	12.6	13.0	16.6	24.0	30.2	39.4	41.6
CO (ppm)	11,770	0.8 ± 0.2	0.6	0.6	0.6	0.7	0.9	1.1	1.3
^***a***^Monthly averages used in 8th month analyses of hypothesis 2.									

**Table 4:** Pearson correlation coefficients for study subject’s 8th-month mean pollutant concentrations and weather parameters.

Variable	Temperature	Relative humidity	PM_2.5_	SO_2_	NO_2_	CO
Temperature	—
Relative humidity	0.85	—
PM_2.5_	0.41	0.28	—
SO_2_	0.10	0.15	0.86	—
NO_2_	–0.96	–0.70	–0.25	0.13	—
CO	0.02	0.23	0.71	0.89	0.20	—

**Figure 3:**
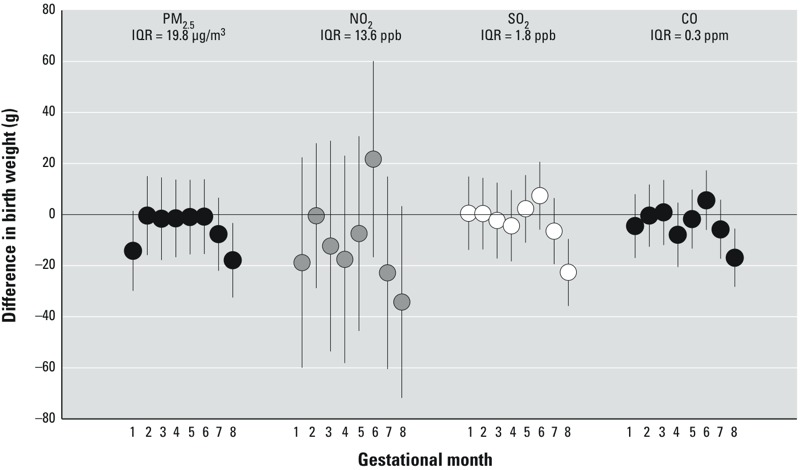
Change in birth weight (g) (95% CI) among term births, associated with each interquartile range (IQR) increase in mean pollutant concentration during a specified month of pregnancy (2 June 2008 to 30 October 2008). All models included indicator variables for gestational age (complete weeks) at delivery, residential district, maternal education (bachelor’s degree, some college or technical school, high school or less), linear terms for the mean temperature and relative humidity levels during the 8th month of pregnancy, and a smooth term for maternal age (smoothing spline with 4 degrees of freedom).

Interquartile range (IQR) increases in PM_2.5_, SO_2_, and CO concentration in the 8th month of pregnancy were associated with significant decreases in birth weight, after adjustment for gestational age at delivery, residential district, maternal education, and mean temperature and relative humidity in the same month [PM_2.5_: –18 g, 95% CI: –32 g, –3 g; SO_2_: –23 g, 95% CI: –36 g, –10 g; CO: –17 g, 95% CI: –28 g, –6 g (Figure 3; see also Supplemental Material, Table S1)]. Each IQR increase in the mean NO_2_ concentration in the 8th month was associated with a similarly sized, albeit nonsignificant, decreased birth weight (–34 g; 95% CI: –72 g, 3 g). IQR increases in other monthly mean pollutant concentrations were generally associated with smaller decreases in birth weight, with some being essentially null (e.g., –1 g to 2 g). None were statistically significant (Figure 3; see also Supplemental Material, Table S1). Although not statistically significant, each 13.6-ppb increase in NO_2_ concentration during the 6th month was associated with a 22-g increase in birth weight (95% CI: –17 g, 60 g). However, 6th-month and 8th-month pollutant concentrations were inversely correlated (PM_2.5_; *r* = –0.71; NO_2_: *r* = –0.95; SO_2_: *r* = –0.88; CO: *r* = –0.70), likely due to one occurring during the Olympics period and its lower air pollutant concentrations.

## Discussion

We took advantage of the natural experiment that occurred during the 2008 Beijing Summer Olympics, where the Chinese government successfully reduced and restricted air pollution emissions in Beijing and the surrounding province, resulting in 20–60% reductions in most pollutants (Rich et al. 2012; Zhang et al. 2013). We examined whether babies born to mothers residing in Beijing during this time had babies with larger birth weights, and whether certain air pollutant concentration reductions were associated with this response. We found that if the 8th month of pregnancy occurred during the 2008 Olympics, the mean birth weight among infants born at term was 23 g larger than those pregnancies with their 8th month during the same dates in 2007 or 2009. This association was independent of maternal age and education, residential district in Beijing, gestational age, and pregnancy-related hypertensive and fetal–placental pregnancy complications. Further, this difference was not limited to women living in just one Beijing district, and was seen in women of all education levels, although the increase in birth weight was largest among women with a bachelor’s degree or more. Similarly, using air pollution measurements made in the center of Beijing from a longer time period, IQR increases in PM_2.5_ (19.8 μg/m^3^), SO_2_ (1.8 ppb), NO_2_ (13.6 ppb), and CO (0.3 ppm) concentrations during the 8th month of pregnancy were associated with –18 g (95% CI: –32 g, 3 g), –23 g (95% CI: –36 g, –10 g), –34 g (95% CI: –72 g, 3 g), and –17 g (95% CI: –28 g, –6 g) decreases in birth weight among term births, respectively. We found no such evidence of consistent associations across our hypothesis 1 and 2 analyses for pollution exposure during months 1–7. These complementary analyses provide evidence that decreases in ambient air pollution levels during the 2008 Summer Olympics and Paralympics, during the 8th month of pregnancy, had a beneficial impact on pregnancies, specifically on birth weight.

These findings are consistent with those of Wang et al. (1997), who reported 10% (95% CI: 5, 14) and 11% (95% CI: 6, 16) increases in the risk of low birth weight associated with 100-μg/m^3^ increases in total suspended particulate and SO_2_ concentrations across the entire pregnancy, respectively, among pregnant women living in some of the same Beijing districts as those in our analysis (Wang et al. 1997). In a meta-analysis of 14 existing studies done around the world, both increased PM_10_ and PM_2.5_ concentrations during the entire pregnancy were associated with an increased risk of low birth weight, with a 10-μg/m^3^ increase in PM_10_ concentration associated with a 8.9-g (95% CI: –13.2 g, –4.6 g) decrease in birth weight (Dadvand et al. 2013). Our findings, rescaled to a 10-μg/m^3^ increase in PM_2.5_ during the 8th month of pregnancy (–9 g; 95% CI: –32 g, –3 g) for comparison, are also consistent with another meta-analysis (some of the same studies), which reported that 20-μg/m^3^ increases in mean PM_10_ concentration and 10-μg/m^3^ increases in mean PM_2.5_ concentrations during the entire pregnancy, were associated with –16.8-g (95% CI: –20.2 g, –13.3 g) and –23.4-g (95% CI: –45.5 g, –1.4 g) decreases in birth weight, respectively (Stieb et al. 2012). Our findings, although for only the 8th month of pregnancy and not the entire pregnancy, are consistent with these meta-analyses.

Our finding of greater birth weight associated with lower air pollution levels during the natural experiment of the Beijing Olympics is consistent with recent work by Fleischer et al. (2014), who reported that each 10-μg/m^3^ increase in PM_2.5_ concentrations in the last month of pregnancy was associated with a significant 7% increase (95% CI: 1, 14) in the risk of a low-birth-weight baby among term births in China (Fleischer et al. 2014). Similarly, our findings are consistent with those of Pedersen et al. (2013), who reported that each 5-μg/m^3^ increase in PM_2.5_ concentration and each 10-μg/m^3^ increase in PM_10_ concentration during pregnancy were associated with increased risks of low birth weight at term [PM_10_: odds ratio (OR) = 1.18, 95% CI: 1.06, 1.33; PM_2.5_: OR = 1.16, 95% CI: 1.00, 1.35]. However, our findings are inconsistent with those of Parker et al. (2008), who reported no beneficial increase in birth weight associated with a Utah Valley steel mill employee strike from August 1986 through September 1987. In studies examining individual trimesters during pregnancy, our findings are consistent with those reporting birth weight associations with increased air pollutant concentrations in the third trimester, but not those reporting associations with first- and second-trimester pollutant exposures (Woodruff et al. 2009).

*Potential mechanistic explanations.* Our finding of increased birth weight associated with decreased pollutant concentrations in the 8th month of pregnancy may suggest potential mechanisms acting during that time of pregnancy, including those mechanisms that affect maternal, placental, or fetal health. Maternal factors associated with poorer fetal growth include maternal hypertension and cardiopulmonary disease. Though exposure to air pollution in pregnancy has been associated with an increased risk of hypertensive disorders later in pregnancy (Hampel et al. 2011; van den Hooven et al. 2011; Wu et al. 2009), we found similar estimates of birth-weight differences associated with having the 8th month of pregnancy during the 2008 Olympics compared with the same dates in 2007 or 2009, for pregnancies with and without hypertensive complications or with and without fetal/placental complications. This suggests that late pregnancy pollutant effects on fetal growth may not be mediated primarily by these complications.

Evidence from observational studies suggests that exposure to PM may affect the human placenta during pregnancy: van den Hooven et al. (2012b) reported that increased ambient PM_10_ and NO_2_ concentrations during pregnancy were associated with higher levels of soluble fms-like tyrosine kinase and lower levels of placental growth factor (anti-angiogenic effects), as well as an increased risk of placental notching during the third trimester (abnormality on ultrasound; marker of blood flow resistance). Janssen et al. (2012) reported that increased PM_10_ concentrations during the last month of pregnancy were associated with decreased placental mitochondrial DNA content, which have been associated with increased oxidative stress and inflammation. Increased maternal C-reactive protein levels have also been associated with increased pollutants concentrations during pregnancy (van den Hooven et al. 2012a). Exposure to air pollution during the 7 days before delivery was associated with lower interleukin (IL)–10 (an anti-inflammatory cytokine) measured in cord blood, and during the 3 months leading up to delivery with higher levels of the inflammatory mediator IL-1β also measured in cord blood (Latzin et al. 2011). Further, in a panel of healthy young men and nonpregnant women living in Beijing before, during, and after the 2008 Summer Olympics, we reported large decreases (–13% to –34%) in markers of oxidative stress, inflammation, and thrombosis during the Olympics when air pollutant concentrations were decreased substantially (–18% to –59%) compared with pre-Olympics levels (Gong et al. 2013; Rich et al. 2012; Zhang et al. 2013). However, whether PM exposure leading to changes in mitochondrial DNA content, inflammation, and oxidative stress in the last month of pregnancy affects placental function and then fetal growth has not yet been studied.

*Strengths and limitations.* Our study had several strengths, including the use of a natural experiment design with its better control of confounding than purely observational designs (Dominici and Mittleman 2012), large sample size, and available air pollutant monitoring data. For the first time, we were able to demonstrate in complementary analyses in the same study that a gestational month (8th month) when pollutant concentrations were decreased was associated with increased birth weight, and increased pollutant concentrations during the same 8th gestational month was associated with decreased birth weight. Isolation of such a short duration time window during pregnancy (i.e., 47 days) where air pollutant concentrations changed substantially and then returned to background levels, to study whether that was associated with a measurable difference in birth weight, has not been possible in previous epidemiologic studies. Previous studies have generally been limited to studying whether trimester-specific mean pollutant concentrations were associated with markers of reduced fetal growth (Dadvand et al. 2013; Rich et al. 2009; Woodruff et al. 2009).

However, there are several limitations that should be considered when making inference from these results. First, for our air pollution analyses, we used ambient PM_2.5_, NO_2_, SO_2_, and CO concentrations, measured at one central-site monitoring location, to represent each pregnancy’s gestational monthly air pollution exposure, regardless of how close the pregnant woman lived, worked, or was near the monitoring site, resulting in exposure error. However, on average, this error is likely a combination of Berkson and classical error (Bateson et al. 2007; Zeger et al. 2000), with any classical error likely resulting in a bias toward the null and underestimates of effect.

Second, although we measured multiple particle ions and species, gases, polycyclic aromatic hydrocarbons, and elemental composition of particles in our previous study (Rich et al. 2012; Zhang et al. 2013), we did not measure these pollutants every day during the study period, resulting in limited variability in monthly mean concentrations in many of them (e.g., benzo[*a*]pyrene, total polycyclic aromatic hydrocarbons). (Zhang et al. 2013). Further, given the drastic pollutant concentration decrease at the beginning of the Olympics period and the increase after the Olympics (Rich et al. 2012; Zhang et al. 2013), these pollutants were all highly correlated during the study period, making assessments of health effects associated with individual pollutants and/or pollutant sources difficult.

This estimated increase in birth weight associated with having the 8th month of pregnancy during the 2008 Olympics period, compared with the same dates in 2007 and 2009, could be due to residual confounding by socioeconomic status. If women of lower socioeconomic status (e.g., lower level of maternal education), who are also at higher risk of adverse pregnancy outcomes (e.g., low birth weight, preterm birth) moved out of the study area during the 2008 Olympics, this could explain the estimated increase in birth weight associated with the 2008 Olympics. However, we do not feel this was the case, because women in 2008 had a larger proportion with a bachelor’s degree (54.7%) than women in 2007 (50.8%). Residual confounding by other factors such as maternal stress and noise associated with living near the Olympics venues or Olympics traffic could also affect the estimated birth weight difference.

We did not have data on several potential confounders, including parity, prepregnancy body mass index, smoking, attendance at prenatal care visits, and race/ethnicity. However, China’s Family Planning Policy (also known as the One-Child Policy), enacted in 1979, restricted the number of children married couples in Beijing could have during the study period to one, with exceptions for those living in rural areas, ethnic minorities, and for couples where each partner had no siblings. Therefore, one can assume that almost all of the pregnancies included in this study are the first child for each pregnant woman. Second, smoking rates in women living in Chinese cities are generally low. A recent letter reported that in 2010, an estimated 28.1% of adults in China (52.9% of men and 2.4% of women) were current smokers, and that the prevalence was significantly higher among rural residents than urban inhabitants (Li et al. 2011), such as those in our study. We did not have data on secondhand smoke exposure of women in our study. However, our hypothesis 1 analyses—temporal analyses comparing the birth weight of pregnancies in 2008 to pregnancies in 2007 and 2009—are unlikely to be confounded by this unless there are substantial differences in secondhand smoke exposure for pregnant women in 2008 versus 2007 and 2009. Third, in Beijing during the study period there was little variability in race/ethnicity, with the vast majority being Han Chinese. Last, gestational age was defined using the date of the last day of the last menstrual period, rather than an ultrasound during pregnancy, resulting in some error. Therefore, this could result in residual confounding by gestational age in our analyses.

This 47-day reduction in ambient air pollutant concentrations during the Beijing Olympics and Paralympics may be too short a time period for beneficial reproductive health effects to be observed if this air pollution reduction occurred early in pregnancy. For example, for pregnancies with their 1st, 2nd, or 3rd months of pregnancy during the Olympics, it is possible that any beneficial fetal growth effect of an Olympics air pollution reduction during these times was then masked by adverse fetal growth effects of air pollution increases in months 4–9 when air pollution returned to the normally elevated levels after the Olympics. Our estimate of a 23-g bigger baby associated with having the 8th month of pregnancy during the Olympics period is a small increase relative to the median birth weight in our study population (< 1%). However, this size increase in birth weight may have been larger if the air pollutant concentration reductions lasted for several months or lasted the entire pregnancy.

We did not have residential addresses during pregnancy, only the residential district at the time of delivery. However, air pollution reductions were observed across Beijing, and as long as subjects remained in Beijing, the substantially lower air pollutant concentrations in the summer of 2008 compared with 2007 and 2009 would be comparable proxies for subjects’ ambient air pollution exposures during these time periods. Because we did not have district-specific air pollutant measurements, we could also not assess whether pollutant/birth weight associations were different for each district.

Last, China’s Family Planning Policy restricted the number of children married couples in Beijing could have during the study period to one, with few exceptions. This likely resulted in healthier pregnancies in this Beijing study population compared with the U.S. population (e.g., fewer pregnancy complications, fewer preterm births, fewer small for gestational age babies), as evidence shows a lower preterm birth rate in Beijing in 2008 (3.3%) compared with that of the United States in 2008 (12.3%) (Martin et al. 2010) and 2012 (11.5%) (Hamilton et al. 2013). Therefore, this may limit the generalizability of our findings to generally healthy pregnancies.

In summary, reductions in late-pregnancy ambient air pollution exposures were associated with increased birth weights among pregnant women living in Beijing during the 2008 Summer Olympics. However, the associations of birth weight with PM_2.5_, SO_2_, and CO concentrations that we report should not suggest associations of individual pollutants with any health effects. Rather, the entire air pollutant mixture or specific components correlated with PM_2.5_, SO_2_, NO_2_, and CO concentrations in Beijing during this time may be responsible for these estimated improvements in birth weight late in pregnancy. Further work should replicate this finding, examine potential mechanistic explanations, and study health effects of specific pollutant mixtures (e.g., traffic pollution, secondary organic aerosols).

## Supplemental Material

(164 KB) PDF

## References

[r1] Barnett AG 2011 Time-dependent exposures and the fixed-cohort bias [Letter]. Environ Health Perspect 119 A422 A423; 10.1289/ehp.1103885 21968256 PMC3230453

[r2] Bateson TF, Coull BA, Hubbell B, Ito K, Jerrett M, Lumley T (2007). Panel discussion review: session three—issues involved in interpretation of epidemiologic analyses—statistical modeling.. J Expo Sci Environ Epidemiol.

[r3] Clancy L, Goodman P, Sinclair H, Dockery DW (2002). Effect of air-pollution control on death rates in Dublin, Ireland: an intervention study.. Lancet.

[r4] Dadvand P Parker J Bell ML Bonzini M Brauer M Darrow LA 2013 Maternal exposure to particulate air pollution and term birth weight: a multi-country evaluation of effect and heterogeneity. Environ Health Perspect 121 267 373; 10.1289/ehp.1205575 23384584 PMC3621183

[r5] Dominici F, Mittleman MA (2012). China’s air quality dilemma: reconciling economic growth with environmental protection.. JAMA.

[r6] Fleischer NL Merialdi M van Donkelaar A Vadillo-Ortega F Martin RV Betran AP 2014 Outdoor air pollution, preterm birth, and low birth weight: analysis of the World Health Organization Global Survey on Maternal and Perinatal Health. Environ Health Perspect 122 425 430; 10.1289/ehp.1306837 24508912 PMC3984219

[r7] Friedman MS, Powell KE, Hutwagner L, Graham LM, Teague WG (2001). Impact of changes in transportation and commuting behaviors during the 1996 Summer Olympic Games in Atlanta on air quality and childhood asthma.. JAMA.

[r8] Gong J, Zhu T, Kipen H, Wang G, Hu M, Ohman-Strickland P (2013). Malondialdehyde in exhaled breath condensate and urine as a biomarker of air pollution induced oxidative stress.. J Expo Sci Environ Epidemiol.

[r9] Hamilton BE, Martin JA, Ventura SJ (2013). Births: preliminary data for 2012.. Natl Vital Stat Rep.

[r10] Hampel R, Lepeule J, Schneider A, Bottagisi S, Charles MA, Ducimetière P (2011). Short-term impact of ambient air pollution and air temperature on blood pressure among pregnant women.. Epidemiology.

[r11] Hastie T, Tibshirani R (1990). Exploring the nature of covariate effects in the proportional hazards model.. Biometrics.

[r12] Hedley AJ, Wong CM, Thach TQ, Ma S, Lam TH, Anderson HR (2002). Cardiorespiratory and all-cause mortality after restrictions on sulphur content of fuel in Hong Kong: an intervention study.. Lancet.

[r13] Heinrich J, Hoelscher B, Wichmann HE (2000). Decline of ambient air pollution and respiratory symptoms in children.. Am J Respir Crit Care Med.

[r14] Janssen BG Munters E Pieters N Smeets K Cox B Cuypers A 2012 Placental mitochondrial DNA content and particulate air pollution during *in utero* life. Environ Health Perspect 120 1346 1352; 10.1289/ehp.1104458 22626541 PMC3440109

[r15] Latzin P Frey U Armann J Kieninger E Fuchs O Röösli M 2011 Exposure to moderate air pollution during late pregnancy and cord blood cytokine secretion in healthy neonates. PLoS One 6 e23130; 10.1371/journal.pone.0023130 21826232 PMC3149643

[r16] Lee JT, Son JY, Cho YS (2007). Benefits of mitigated ambient air quality due to transportation control on childhood asthma hospitalization during the 2002 Summer Asian Games in Busan, Korea.. J Air Waste Manag Assoc.

[r17] Li Q, Hsia J, Yang G (2011). Prevalence of smoking in China in 2010.. N Engl J Med.

[r18] Martin JA, Osterman MJK, Sutton PD (2010). Are preterm births on the decline in the United States? Recent data from the National Vital Statistics System.. NCHS Data Brief.

[r19] Parker JD, Mendola P, Woodruff TJ (2008). Preterm birth after the Utah Valley Steel Mill closure: a natural experiment.. Epidemiology.

[r20] Pedersen M, Giorgis-Allemand L, Bernard C, Aguilera I, Andersen AM, Ballester F (2013). Ambient air pollution and low birth weight: a European cohort study (ESCAPE).. Lancet Respir Med.

[r21] Pope CA (1989). Respiratory disease associated with community air pollution and a steel mill, Utah Valley.. Am J Public Health.

[r22] Pope CA (1991). Respiratory hospital admissions associated with PM_10_ pollution in Utah, Salt Lake, and Cache valleys.. Arch Environ Health.

[r23] Pope CA III Rodermund DL Gee MM 2007 Mortality effects of a copper smelter strike and reduced ambient sulfate particulate matter air pollution. Environ Health Perspect 115 679 683; 10.1289/ehp.9762 17520052 PMC1867960

[r24] R Core Team. (2014). R: A Language and Environment for Statistical Computing. Version 3.1.1 Vienna:R Foundation for Statistical Computing.. http://www.R-project.org.

[r25] Rich DQ, Demissie K, Lu SE, Kamat L, Wartenberg D, Rhoads GG (2009). Ambient air pollutant concentrations during pregnancy and the risk of fetal growth restriction.. J Epidemiol Community Health.

[r26] Rich DQ, Kipen HM, Huang W, Wang G, Wang Y, Zhu P (2012). Association between changes in air pollution levels during the Beijing Olympics and biomarkers of inflammation and thrombosis in healthy young adults.. JAMA.

[r27] Stieb DM, Chen L, Eshoul M, Judek S (2012). Ambient air pollution, birth weight and preterm birth: a systematic review and meta-analysis.. Environ Res.

[r28] Sun Z, Mu Y, Liu Y, Shao L (2013). A comparison study on airborne particles during haze days and non-haze days in Beijing.. Sci Total Environ.

[r29] Tao M, Chen L, Wang Z, Ma P, Tao J, Jia S (2014). A study of urban pollution and haze clouds over northern China during the dusty season based on satellite and surface observations.. Atmos Environ.

[r30] Tonne C, Beevers S, Armstrong B, Kelly F, Wilkinson P (2008). Air pollution and mortality benefits of the London Congestion Charge: spatial and socioeconomic inequalities.. Occup Environ Med.

[r31] van den Hooven EH, de Kluizenaar Y, Pierik FH, Hofman A, van Ratingen SW, Zandveld PY (2011). Air pollution, blood pressure, and the risk of hypertensive complications during pregnancy: the Generation R Study.. Hypertension.

[r32] van den Hooven EH de Kluizenaar Y Pierik FH Hofman A van Ratingen SW Zandveld PY 2012a Chronic air pollution exposure during pregnancy and maternal and fetal C-reactive protein levels: the Generation R Study. Environ Health Perspect 120 746 751; 10.1289/ehp.1104345 22306530 PMC3346784

[r33] van den Hooven EH Pierik FH de Kluizenaar Y Hofman A van Ratingen SW Zandveld PY 2012b Air pollution exposure and markers of placental growth and function: the Generation R Study. Environ Health Perspect 120 1753 1759; 10.1289/ehp.1204918 22922820 PMC3548279

[r34] Wang T, Nie W, Gao J, Xue LK, Gao XM, Wang XF (2010). Air quality during the 2008 Beijing Olympics: secondary pollutants and regional impact.. Atmos Chem Phys.

[r35] Wang X, Ding H, Ryan L, Xu X (1997). Association between air pollution and low birth weight: a community-based study.. Environ Health Perspect.

[r36] Woodruff TJ, Parker JD, Darrow LA, Slama R, Bell ML, Choi H (2009). Methodological issues in studies of air pollution and reproductive health.. Environ Res.

[r37] Wu J Ren C Delfino RJ Chung J Wilhelm M Ritz B 2009 Association between local traffic-generated air pollution and preeclampsia and preterm delivery in the South Coast Air Basin of California. Environ Health Perspect 117 1773 1779; 10.1289/ehp.0800334 20049131 PMC2801174

[r38] Xu DQ, Zhang WL (2004). Monitoring of pollution of air fine particles (PM2.5) and study on their genetic toxicity.. Biomed Environ Sci.

[r39] Zeger SL, Thomas D, Dominici F, Samet JM, Schwartz J, Dockery D (2000). Exposure measurement error in time-series studies of air pollution: concepts and consequences.. Environ Health Perspect.

[r40] Zhang J, Mauzerall DL, Zhu T, Liang S, Ezzati M, Remais JV (2010). Environmental health in China: progress towards clean air and safe water.. Lancet.

[r41] Zhang J, Zhu T, Kipen H, Wang G, Huang W, Rich D (2013). Cardiorespiratory biomarker responses in healthy young adults to drastic air quality changes surrounding the 2008 Beijing Olympics.. Res Rep Health Eff Inst.

[r42] Zhao XJ, Zhao PS, Xu J, Meng W, Pu WW, Dong F (2013). Analysis of a winter regional haze event and its formation mechanism in the North China Plain.. Atmos Chem Phys.

[r43] Zhou Y, Wu Y, Yang L, Fu LX, He KB, Wang SX (2010). The impact of transportation control measures on emission reductions during the 2008 Olympic Games in Beijing, China.. Atmos Environ.

