# Supplementary material for: Differences in Birth Weight Associated with the 2008 Beijing Olympics Air Pollution Reduction: Results from a Natural Experiment
Source: Environ Health Perspect. 2015 Apr 28;123(9):880–7. doi: 10.1289/ehp.1408795 (PMC4559955; doi:10.1289/ehp.1408795)
Supplement: (164 KB) PDF [file ehp.1408795.s001.acco.pdf]

Environ Health Perspect
doi: 10.1289/ehp.1408795

Note to Readers: *EHP* strives to ensure that all journal content is accessible to all readers. However, some figures and Supplemental Material published in *EHP* articles may not conform to 508 standards due to the complexity of the information being presented. If you need assistance accessing journal content, please contact ehp508@niehs.nih.gov. Our staff will work with you to assess and meet your accessibility needs within 3 working days.

Supplemental Material

Differences in Birth Weight Associated with the 2008 Beijing Olympic Air Pollution Reduction: Results from a Natural Experiment

David Q. Rich, Kaibo Liu, Jinliang Zhang, Sally W. Thurston, Timothy P. Stevens, Ying Pan, Cathleen Kane, Barry Weinberger, Pamela Ohman-Strickland, Tracey J. Woodruff, Xiaoli Duan, Vanessa Assibey-Mensah, and Junfeng Zhang

Table of Contents

Table 1. Change in birth weight (g; term births), associated with each interquartile range (IQR) increase in mean pollutant concentration during each gestational month.

Table 1. Change in birth weight (g; term births), associated with each interquartile range (IQR) increase in mean pollutant concentration during each gestational month.

	$PM_{2.5}$ IQR = 19.8 µg/m^3			NO_2 IQR = 13.6 ppb			SO_2 IQR = 1.8 ppb			CO IQR = 0.3 ppm		
**Month**	**N**	**Change in birth weight (g) (95% CI)**	**p-value**	**N**	**Change in birth weight (g) (95% CI)**	**p-value**	**N**	**Change in birth weight (g) (95% CI)**	**p-value**	**N**	**Change in birth weight (g) (95% CI)**	**p-value**
1	10,085	-14 (-30, 1)	0.08	10,778	-19 (-60, 22)	0.37	8,186	1 (-14, 15)	0.94	10,778	-5 (-17, 8)	0.48
2	9,502	0.0 (-16, 15)	0.96	10,556	0 (-29, 28)	0.30	8,562	0 (-14, 14)	0.95	10,557	0 (-13, 12)	0.95
3	9,589	-2 (-18, 15)	0.84	10,652	-12 (-54, 29)	0.56	7,808	-2 (-17, 13)	0.76	10,653	1 (-12, 14)	0.90
4	10,158	-1 (-17, 14)	0.85	12,361	-18 (-58, 23)	0.40	8,274	-4 (-18, 10)	0.54	11,273	-8 (-21, 5)	0.22
5	11,177	-1 (-16, 14)	0.90	11,273	-7 (-46, 31)	0.70	9,258	2 (-11, 16)	0.74	12,361	-2 (-13, 10)	0.76
6	11,276	-1 (-15, 14)	0.91	12,341	22 (-17, 60)	0.27	9,180	7 (-6, 21)	0.27	12,342	6 (-6, 17)	0.34
7	11,114	-8 (-22, 7)	0.29	12,033	-23 (-60, 15)	0.24	9,116	-7 (-19, 7)	0.33	12,033	-6 (-17, 6)	0.33
8	10,771	-18 (-32, -3)	0.02	11,770	-34 (-72, 3)	0.07	8961	-23 (-36, -10)	<0.001	11,770	-17 (-28, -6)	0.004

All models included indicator variables for gestational age (complete weeks) at delivery, residential district, maternal education (bachelor's degree, some college or technical school, high school or less), linear terms for the mean temperature and relative humidity levels during the same 1st month of pregnancy, and a smooth term for maternal age (smoothing spline with 4 degrees of freedom).
